# Tapinarof cream for the treatment of plaque psoriasis: Efficacy and safety results from 2 Japanese phase 3 trials

**DOI:** 10.1111/1346-8138.17423

**Published:** 2024-08-16

**Authors:** Atsuyuki Igarashi, Gaku Tsuji, Shuichi Fukasawa, Ryusei Murata, Satoshi Yamane

**Affiliations:** ^1^ Igarashi Dermatology Higashigotanda Tokyo Japan; ^2^ Research and Clinical Center for Yusho and Dioxin Kyushu University Fukuoka Japan; ^3^ Department of Dermatology, Graduate School of Medical Sciences Kyushu University Fukuoka Japan; ^4^ Japan Tobacco Inc. Tokyo Japan

**Keywords:** aryl hydrocarbon receptor, clinical trial, phase 3, plaque psoriasis, tapinarof

## Abstract

Tapinarof is a non‐steroidal, topical, aryl hydrocarbon receptor agonist. We evaluated the efficacy and safety of tapinarof cream (1%) in Japanese patients aged ≥18 years with plaque psoriasis in two phase 3 trials, ZBA4‐1 and ZBA4‐2. ZBA4‐1 (*N* = 158) consisted of a 12‐week, double‐blind, vehicle‐controlled treatment period (period 1) and a 12‐week extension treatment period (period 2). Patients were randomized 2:1 to tapinarof or vehicle in period 1; subsequently, all patients who were enrolled in period 2 received tapinarof. ZBA4‐2 (*N* = 305) was a 52‐week, open‐label, uncontrolled trial in which all patients received tapinarof. In period 1 of ZBA4‐1, the proportion of patients who achieved a Physician Global Assessment (PGA) score of 0 (clear) or 1 (almost clear) with ≥2‐grade improvement from baseline at week 12 (PGA treatment success, the primary endpoint) was 20.06% in the tapinarof group and 2.50% in the vehicle group (*p* = 0.0035). The proportion of patients with ≥75% improvement from baseline in the Psoriasis Area and Severity Index (PASI) score at week 12 (PASI75 response, a key secondary endpoint) was 37.7% in the tapinarof group and 3.8% in the vehicle group (*p* < 0.0001). In ZBA4‐2, PGA treatment success rate was 30.0% at week 12, 51.3% at week 24, and 56.3% at week 52, and PASI75 response rate was 50.4% at week 12, 77.5% at week 24, and 79.9% at week 52, indicating that efficacy responses improved over time and were maintained over 52 weeks. Across the two trials, most adverse events (AEs) were mild or moderate; common AEs included folliculitis and contact dermatitis. In summary, tapinarof cream (1%) was efficacious and generally safe for up to 52 weeks of treatment in Japanese patients with plaque psoriasis.

## 1 INTRODUCTION

Plaque psoriasis is a chronic, recurrent skin disease that results from multiple causes, including genetic, environmental, and immunopathological factors.^1^ The primary manifestation of plaque psoriasis is well‐circumscribed erythema and erythematous plaques covered with silver scales.^2^ Psoriatic lesions contain increased numbers of activated T cells, which have an important role in the pathogenesis of inflammatory diseases through the abnormal secretion of pro‐inflammatory cytokines (e.g., interleukin [IL]‐17A, IL‐23, and tumor necrosis factor α).^2^ Psoriasis has a substantial impact on the quality of life (QOL) of patients and is associated with reduced productivity and increased medical costs.^3^ The prevalence of psoriasis varies among countries, ranging from 0.91% to 8.5% in western countries,^4^ while in Japan it is estimated to be 0.34%.^5^ Psoriasis in Japan is more common in men than in women, with a ratio of approximately 2:1.^6^ Topical therapies are still the mainstay of treatment for most patients with psoriasis across a wide range of disease severity. Such therapies are utilized even after systemic therapies, including biologics, have become available for the treatment of moderate‐to‐severe psoriasis.^7^, ^8^ Current topical therapies, such as corticosteroids, have some restrictions on sites of application, amount of application, and duration of treatment because of potential adverse reactions and loss of efficacy.^8^ Thus, there remains a need for efficacious topical therapies with an acceptable safety profile that can be used on a large body surface area (BSA) and for long‐term disease control.

Tapinarof is a non‐steroidal, topical, aryl hydrocarbon receptor (AhR) agonist,^9^ which has been approved by the US Food and Drug administration for the treatment of plaque psoriasis.^10^, ^11^ Tapinarof directly binds to and activates AhR, leading to downregulated expression of pro‐inflammatory cytokines, including IL‐17A and IL‐17F, and upregulated expression of skin barrier proteins.^9^ Tapinarof also activates the nuclear factor erythroid 2‐related factor 2 pathway, leading to upregulated expression of antioxidant enzyme genes.^9^, ^12^ These findings indicate that the efficacy of tapinarof in psoriasis can be attributed to anti‐inflammatory, skin barrier function improvement, and antioxidant effects.

The results of a global phase 2 trial in patients with plaque psoriasis showed that tapinarof cream (1%) was efficacious in the Japanese subpopulation as well as the overall population.^13^, ^14^ Here, we describe the efficacy and safety of tapinarof cream (1%) in patients with plaque psoriasis from two Japanese phase 3 trials, ZBA4‐1 and ZBA4‐2.

## 2 METHODS

### 2.1 Trial design

**FIGURE 1:**
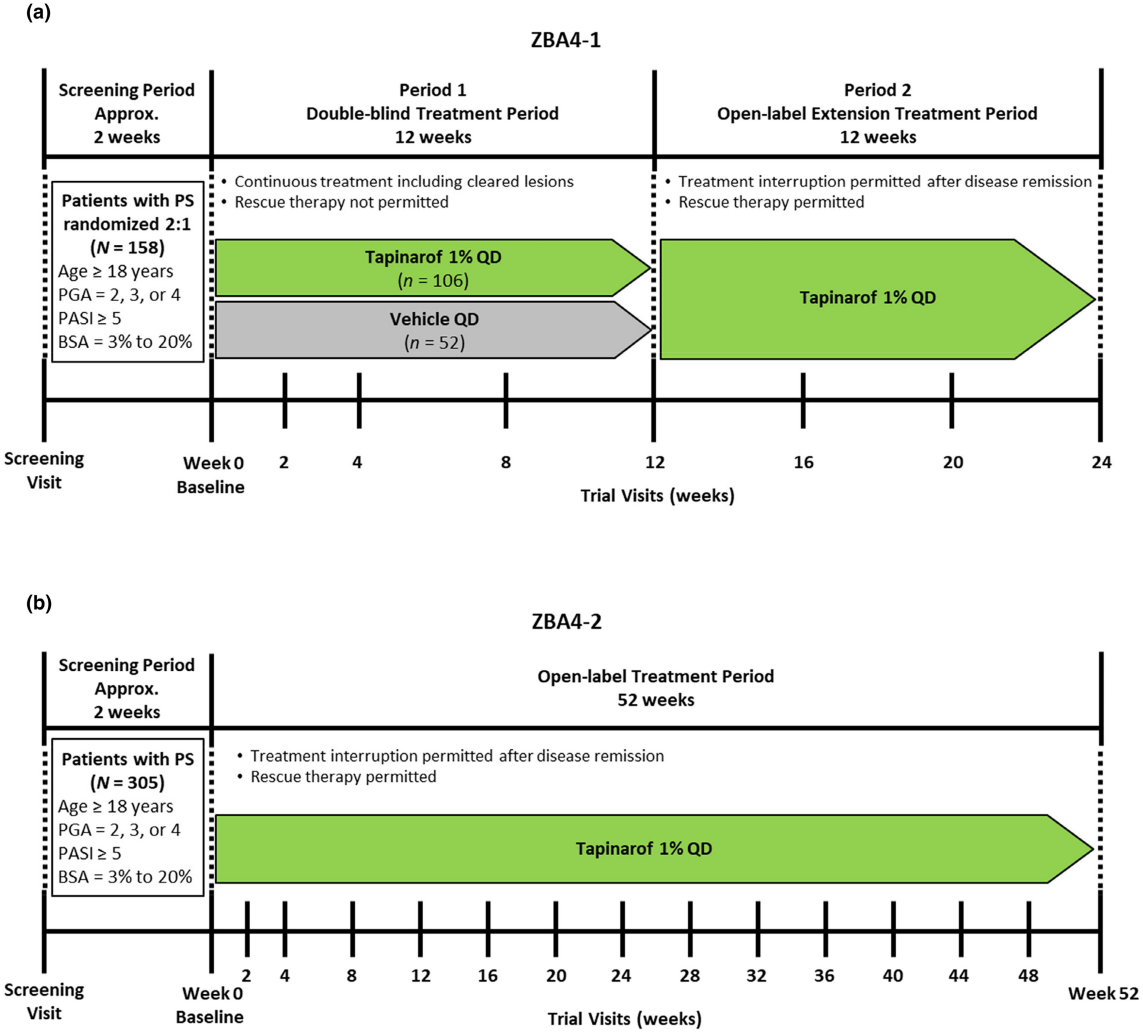
Trial design. (a) ZBA4‐1. (b) ZBA4‐2. BSA, body surface area; PASI, Psoriasis Area and Severity Index; PGA, Physician Global Assessment; PS, plaque psoriasis; QD, once daily.

Trial ZBA4‐1 was conducted at 33 sites in Japan, consisting of a 12‐week, double‐blind treatment period (period 1) and a 12‐week extension treatment period (period 2) (Figure 1). Period 1 was a randomized, double‐blind, vehicle‐controlled trial in which patients were randomized 2:1 to tapinarof cream (1%) or vehicle cream. A computer‐generated randomization was performed with a dynamic allocation method to balance for the Physician Global Assessment (PGA) score.^15^, ^16^, ^17^ Patients who completed period 1 had the option to enroll in period 2. Period 2 was an open‐label, uncontrolled trial in which all patients received tapinarof cream (1%). Trial visits after the initiation of trial treatment occurred at weeks 2, 4, and every 4 weeks thereafter up to week 24.

ZBA4‐2 was conducted at 57 trial sites in Japan and was a 52‐week, open‐label, uncontrolled trial in which all patients received tapinarof cream (1%) (Figure 1). Trial visits after initiation of the trial treatment occurred at weeks 2, 4, and every 4 weeks thereafter up to week 52.

Both trials were conducted in compliance with the guidelines for Good Clinical Practice and the Declaration of Helsinki. The protocols were approved by the institutional review board at each trial site. Written informed consent was provided by the patients or the legal guardians of patients aged <20 years.

### 2.2 Patients

In both trials, eligible Japanese patients were aged ≥18 years with a clinical diagnosis of chronic plaque psoriasis, stable for ≥6 months before the trials. They had a PGA score of 2 (mild), 3 (moderate), or 4 (severe); a %BSA affected of ≥3% to ≤ 20% (except for the hairy scalp), and a Psoriasis Area and Severity Index (PASI) score^16^, ^17^ of ≥5 (except for the hairy scalp). Both trials excluded patients with a significant dermatological or inflammatory condition that would make it difficult to interpret data or evaluations during the trials, an acute active skin infection, or use of therapies for psoriasis within the indicated period before baseline (e.g., phototherapy, systemic corticosteroids, and immunosuppressive agents within 4 weeks, topical corticosteroids within 2 weeks or 1 week; Table S1).

### 2.3 Trial treatment

Patients were instructed to apply a thin layer of trial treatment once daily to all psoriasis lesions (except for the hairy scalp), including newly appearing lesions. If lesions had cleared, application to the areas was to be continued in period 1 of ZBA4‐1 but could be interrupted at the investigator's discretion in period 2 of ZBA4‐1 and in ZBA4‐2.

Therapies that were indicated for psoriasis or that may have been effective in psoriasis were prohibited during the trials (e.g., phototherapy, systemic corticosteroids, immunosuppressive agents, topical corticosteroids; Table S1). In period 2 of ZBA4‐1 and in ZBA4‐2, however, these therapies could be used at the investigator's discretion as rescue therapy if the psoriasis lesions had not improved.

### 2.4 Trial assessments

The PGA, PASI, and %BSA affected assessments were performed by the investigators at each trial visit. The 5‐point PGA score, ranging from 0 (clear) to 4 (severe), was used. The pruritus numeric rating scale (NRS) score,^18^, ^19^ ranging from 0 (no itch) to 10 (worst itch imaginable), was recorded daily in a diary by patients in period 1 of ZBA4‐1 and was assessed at each trial visit by patients in period 2 of ZBA4‐1 and in ZBA4‐2. The Skindex‐16 questionnaire^20^ for assessing the impact of psoriasis on QOL was completed by patients at selected trial visits.

In period 1 of ZBA4‐1, the primary endpoint was the proportion of patients who achieved PGA treatment success, defined as a PGA score of 0 (clear) or 1 (almost clear) with ≥2‐grade improvement from baseline at week 12. Key secondary endpoints were the proportion of patients with ≥75% improvement from baseline in PASI score (PASI75) and the proportion of patients with a PGA score of 0 or 1 at week 12. Other secondary endpoints included the mean percentage change from baseline in PASI score, the proportion of patients with ≥50% and ≥90% improvement from baseline in PASI score (PASI50 and PASI90, respectively), the mean change from baseline in %BSA affected, the proportion of patients achieving ≥3‐point and ≥4‐point improvement from baseline in pruritus NRS score, and the mean change from baseline in Skindex‐16 score. Across periods 1 and 2 (overall trial period) of ZBA4‐1 and in ZBA4‐2, most of the above endpoints were included for long‐term efficacy assessments.

Safety assessments included the incidence and severity of adverse events (AEs), clinical laboratory parameters, and vital signs. Concentrations of tapinarof were determined in plasma samples collected at selected visits.

### 2.5 Statistical analyses

For ZBA4‐1, the sample size was calculated on the basis of the results of the phase 2 trial of tapinarof in patients with plaque psoriasis.^13^, ^14^ A sample size of 108 patients (72 patients in the tapinarof group, 36 patients in the vehicle group) would provide an at least 90% power for a statistical significance at the 5% significance level with a 2‐sided Fisher's exact test. This was assuming that the PGA treatment success rate at week 12, the primary endpoint, would be 40% in the tapinarof group and 10% in the vehicle group. The target sample size was determined to be 150 patients (100 patients in the tapinarof group, 50 patients in the vehicle group), assuming that approximately 25% of the patients would be prematurely discontinued from the trial by week 12. For ZBA4‐2, no formal statistical calculation of the sample size was performed because it was an open‐label, uncontrolled trial.

For period 1 of ZBA4‐1, the primary analyses of efficacy were performed in the full analysis set (FAS), which consisted of all randomized patients who received at least one application of the trial treatment and underwent a PGA at least once. The primary endpoint (PGA treatment success rate at week 12) was analyzed based on 100 datasets, where missing data were imputed by the multiple imputation (MI) with the fully conditional specification (FCS) model. The FCS model successively imputed missing data from week 2 to week 12, using linear regression models including the treatment group, baseline value, and post baseline values as covariates. The Cochran–Mantel–Haenszel test stratified by baseline PGA score (2,3, or 4) was performed at the 2‐sided significance level of 5%. The common between‐group difference in the rate and its 95% confidence interval (CI) (Sato method^21^) was calculated by combining the between‐group differences in each baseline PGA score stratum. The key secondary endpoints (rates of PASI‐75 response and patients with a PGA score of 0 or 1 at week 12) were analyzed based on the first dataset out of 100 datasets where missing data were imputed by the MI. The Fisher's exact test was performed at the 2‐sided significance level of 5%. The between‐group difference in the rate and its 95% CI (Exact) were calculated. Other secondary endpoints were analyzed based on data imputed by the MI or on observed cases (OC) where missing data were not imputed; no formal statistical tests were planned for comparisons between the groups. No adjustment was performed for multiple comparisons.

For the overall trial period of ZBA4‐1 and ZBA4‐2, the efficacy analyses were performed in the efficacy analysis population for each trial. This analysis included all patients who received at least one application of tapinarof cream (1%) and who underwent the efficacy assessment at least once. Thus, for the overall trial period of ZBA4‐1, vehicle‐treated patients who were discontinued from the trial in period 1 (by week 12) were excluded from the analyses. Efficacy endpoints were analyzed based on OC where missing data were not imputed.

The safety analyses were performed in the safety analysis population for each trial, which consisted of all patients who received at least one application of trial treatment for period 1 of ZBA4‐1 and received at least 1 application of tapinarof cream (1%) for the overall trial period of ZBA4‐1 and ZBA4‐2. Pooled safety analyses were also performed with data from ZBA4‐1 and ZBA4‐2 as well as Japanese patients in the tapinarof cream (1%) once daily group of the phase 2 trial (six patients). Verbatim terms of AEs reported by the investigators were coded according to the Medical Dictionary for Regulatory Activities, version 24.0.

Pharmacokinetic analyses were performed in the pharmacokinetic analysis population for each trial, which consisted of all patients who had plasma concentration data of tapinarof (including plasma concentration below the lower limit of quantification [LLOQ; 50.0 pg/mL]) at ≥1 time point.

## 3 RESULTS

### 3.1 Patients

**FIGURE 2:**
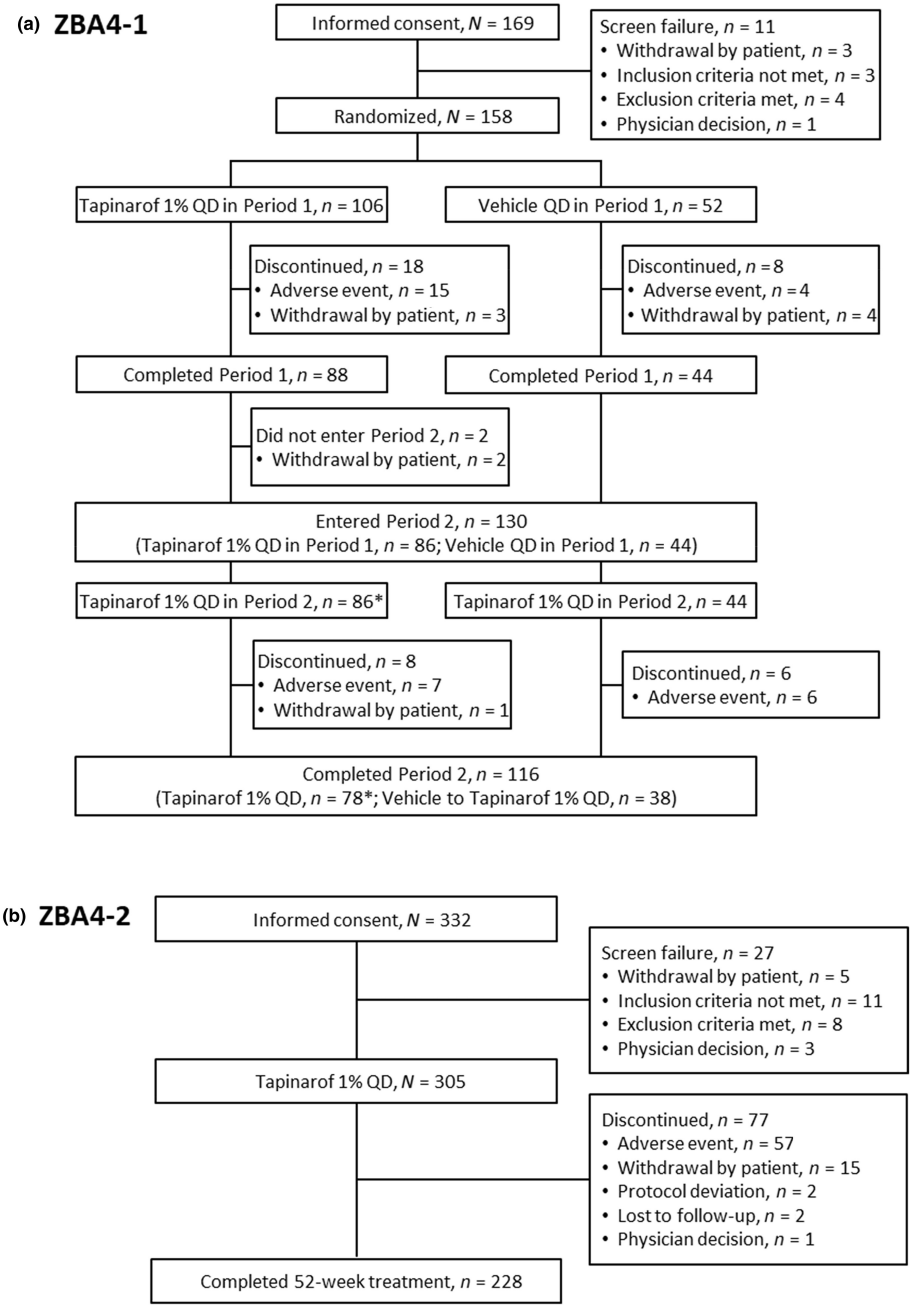
Patient disposition. (a) ZBA4‐1. (b) ZBA4‐2. QD, once daily. *Including 2 patients in ZBA4‐1 who did not receive tapinarof throughout period 2 because of disease remission.

In ZBA4‐1, of 158 randomized patients, 106 received tapinarof and 52 received the vehicle in period 1. Of 132 patients who completed period 1, 86 continued the treatment with tapinarof, while 44 switched from vehicle to tapinarof in period 2. A total of 116 patients completed period 2. In ZBA4‐2, of 305 patients who received tapinarof, 228 completed the trial. Overall, the common reasons for trial discontinuation included AEs and withdrawal by patients in both trials (Figure 2).

In period 1 of ZBA4‐1, no apparent differences between the treatment groups were noted in the demographics and baseline disease characteristics. In both treatment groups, approximately 80% of patients had a baseline PGA score of 3 (moderate). In ZBA4‐2, approximately 60% of patients had a baseline PGA score of 3 (Table 1 and Table S2).

**TABLE 1:** Patient demographics and baseline disease characteristics.

	ZBA4‐1 period 1 (double‐blind)^a^		ZBA4‐2^b^ (*n* = 304)
	Tapinarof 1% (*n* = 106)	Vehicle (*n* = 52)	
Age, mean (SD), years	53.1 (14.0)	60.8 (13.7)	53.3 (13.7)
Male, *n* (%)	79 (74.5)	46 (88.5)	202 (66.4)
Weight, mean (SD), kg	71.1 (15.5)	69.0 (12.3)	69.4 (14.7)
BMI, mean (SD), kg/m^2^	25.4 (4.1)	24.6 (3.4)	25.1 (4.3)
Disease duration, mean (SD), years	13.2 (10.7)	14.2 (9.1)	12.5 (11.8)
PGA score, *n* (%)			
2: mild	17 (16.0)	7 (13.5)	88 (28.9)
3: moderate	84 (79.2)	42 (80.8)	196 (64.5)
4: severe	5 (4.7)	3 (5.8)	20 (6.6)
PASI score, mean (SD)	10.5 (4.1)	11.1 (4.2)	9.6 (3.7)
BSA affected, mean (SD), %	11.7 (5.2)	12.2 (5.6)	11.8 (5.1)
Pruritus NRS score, mean (SD)^c^	3.9 (2.3)	3.6 (2.4)	4.6 (2.7)
Skindex‐16 total score, mean (SD)	38.6 (20.4)	34.6 (20.8)	40.2 (20.7)

*Note*: Patient demographics and baseline disease characteristics in the overall trial period of ZBA4‐1 and in the pooled safety population are provided in Table S2.

Abbreviations: BMI, body mass index; BSA, body surface area; NRS, Numeric Rating Scale; PASI, Psoriasis Area and Severity Index; PGA, Physician Global Assessment; SD, standard deviation.

^a^ Data based on the full analysis set (FAS).

^b^ Data based on the efficacy analysis population.

^c^ The baseline value for pruritus NRS score in ZBA4‐1 was defined as the mean value of daily scores obtained during the 7 days prior to the initiation of trial treatment (Day −7 to Day −1).

In the efficacy analysis populations, rescue therapy (medication) was used at least once by five of 106 patients (4.7%) in the tapinarof group of ZBA4‐1, eight of 44 patients (18.2%) in the vehicle to tapinarof group of ZBA4‐1, and 94 of 304 patients (30.9%) in ZBA4‐2. The most common rescue therapy was topical corticosteroids in both trials (Table S3).

### 3.2 Efficacy

**FIGURE 3:**
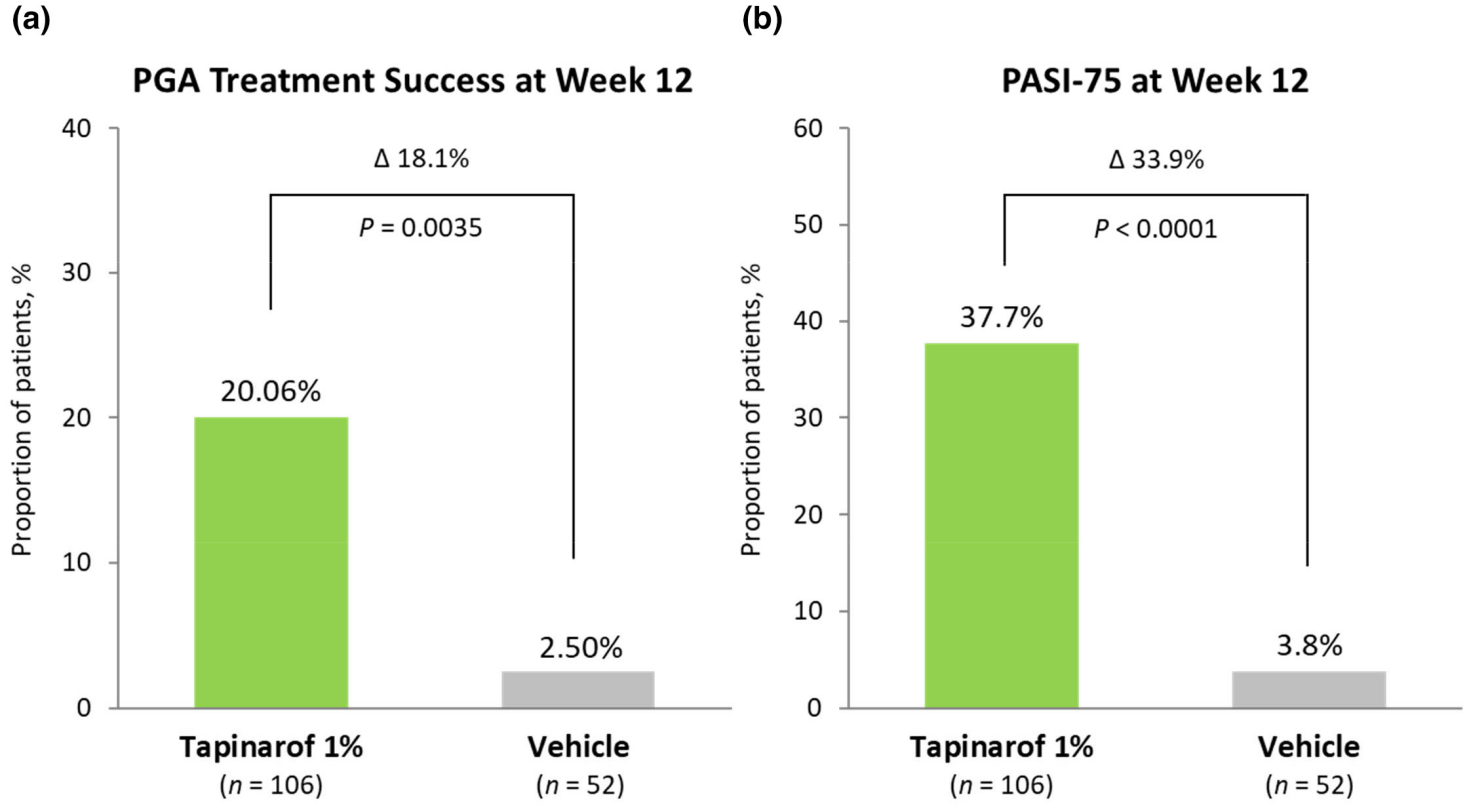
Primary and key secondary endpoints in period 1 of ZBA4‐1. (a) Physician Global Assessment (PGA) treatment success rate at week 12, (b) Psoriasis Area and Severity Index (PASI)75 response rate at week 12. PGA treatment success was defined as a PGA score of 0 or 1 with ≥2‐grade improvement from baseline and was analyzed based on 100 datasets where missing data were imputed by multiple imputation (MI) model. PASI75 was defined as ≥75% improvement from baseline in PASI score and was analyzed based on the first dataset out of 100 datasets where missing data were imputed by the MI model.

In period 1 of ZBA4‐1, significant differences between the tapinarof and vehicle groups were noted in the primary and key secondary endpoints. The primary endpoint (PGA treatment success rate at week 12) was 20.06% in the tapinarof group and 2.50% in the vehicle group, with a between‐group difference of 18.1% (95% CI, 8.3%–27.9%; *p* = 0.0035) (Figure 3). Representative clinical images of patients who achieved the PGA treatment success are presented in Figure S1. A key secondary endpoint, PASI75 response rate at week 12, was 37.7% in the tapinarof group and 3.8% in the vehicle group, with a between‐group difference of 33.9% (95% CI, 21.3%–44.5%; *p* < 0.0001) (Figure 3). The other key secondary endpoint, the proportion of patients with a PGA score of 0 or 1 at week 12, was 30.2% in the tapinarof group and 1.9% in the vehicle group, with a between‐group difference of 28.3% (95% CI, 17.1%–38.3%; *p* < 0.0001). For other secondary endpoints, such as %BSA affected, pruritus NRS score, and Skindex‐16 score, greater improvements were noted in the tapinarof group (Table S4 and Figure S2).

In the overall trial period of ZBA4‐1, PGA treatment success and PASI75 response rates in the tapinarof group continued to increase through week 24. The PGA treatment success rate was 19.0% at week 12 and 58.4% at week 24; the PASI75 response rate was 42.2% at week 12 and 80.5% at week 24. In the vehicle to tapinarof group, PGA treatment success and PASI75 response rates increased rapidly after switching to tapinarof at week 12. The PGA treatment success rate was 2.3% at week 12 and 45.9% at week 24. The PASI75 response rate was 4.7% at week 12 and 64.9% at week 24 (Figure S3). Similar results were obtained for the other endpoints (Table S5).

**FIGURE 4:**
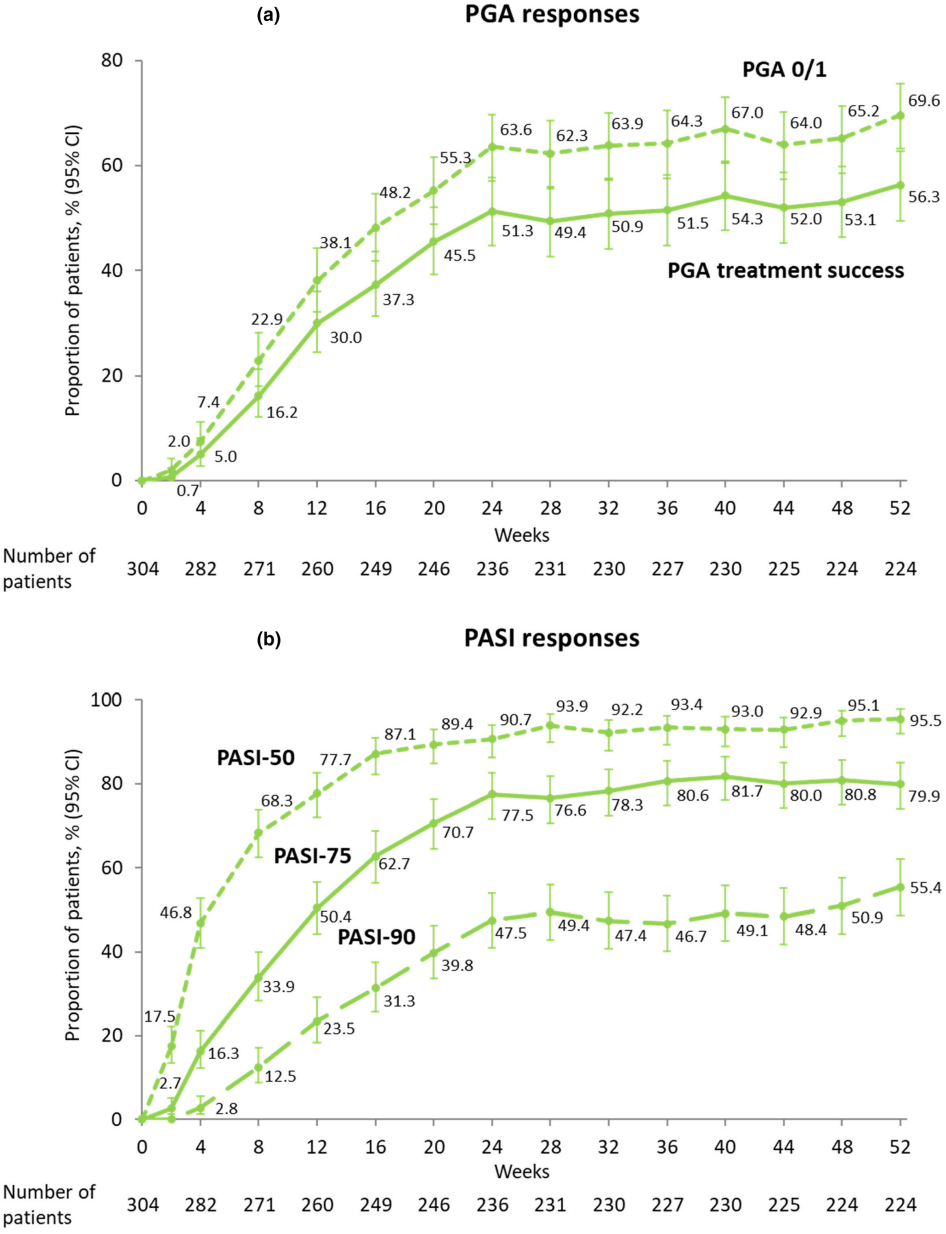
Proportion of patients with (a) a Physician Global Assessment (PGA) treatment success and PGA score of 0 or 1; (b) Psoriasis Area and Severity Index (PASI) response of 50, 75, and 90 in ZBA4‐2. PGA treatment success was defined as a PGA score of 0 or 1 with ≥2‐grade improvement from baseline. PASI50, 75, and 90 were defined as ≥50%, ≥ 75%, and≥ 90% improvement from baseline in PASI score, respectively. Data were analyzed based on observed cases (OC) where missing data were not imputed and are presented with exact 95% confidence intervals (CIs).

In ZBA4‐2, response rates for PGA score (PGA treatment success, PGA score of 0 or 1) and PASI score (PASI50, PASI75, PASI90) increased over time, and the responses were maintained through week 52 (Figure 4). The PGA treatment success rate was 30.0% at week 12, 51.3% at week 24, and 56.3% at week 52, and The PASI75 response rate was 50.4% at week 12, 77.5% at week 24, and 79.9% at week 52. Similarly, improvements in the other efficacy endpoints were maintained through week 52 (Table S5).

### 3.3 Safety

In period 1 of ZBA4‐1, AEs were reported in 66 of 106 (62.3%) patients in the tapinarof group and in 22 of 52 (42.3%) patients in the vehicle group (Table 2). No serious or severe AE was reported in either group. The most common AEs included contact dermatitis (12.3%), folliculitis (11.3%), and application site folliculitis (10.4%) in the tapinarof group and psoriasis (15.4%, mostly reported as worsening of psoriasis) in the vehicle group. Treatment‐related AEs were reported in 35.8% of patients in the tapinarof group and in 11.5% of patients in the vehicle group. The most common treatment‐related AEs included contact dermatitis (10.4%), application site folliculitis (8.5%), and folliculitis (6.6%) in the tapinarof group and psoriasis (9.6%) in the vehicle group. Trial discontinuations due to AEs occurred in 14.2% of patients in the tapinarof group and in 7.7% in the vehicle group, the majority of which were contact dermatitis (5.7% for tapinarof, 0% for vehicle) and psoriasis (5.7% for tapinarof, 7.7% for vehicle). A summary of AEs in the overall trial periods of ZBA4‐1 and in ZBA4‐2 is provided in Table S6.

**TABLE 2:** Summary of adverse events.

	ZBA4‐1 period 1 (double‐blind)		Pooled safety population^a^ (*n* = 461)
	Tapinarof 1% (*n* = 106)	Vehicle (*n* = 52)	
Any AEs	66 (62.3)	22 (42.3)	384 (83.3)
Serious AEs^b^	0	0	9 (2.0)
Severe AEs^b^	0	0	7 (1.5)
Treatment‐related AEs	38 (35.8)	6 (11.5)	235 (51.0)
AEs leading to discontinuation	15 (14.2)	4 (7.7)	84 (18.2)
Most common AEs (occurring in ≥5% of patients in the pooled safety population)			
Contact dermatitis	13 (12.3)	0	83 (18.0)
Application site folliculitis	11 (10.4)	0	81 (17.6)
Psoriasis	7 (6.6)	8 (15.4)	60 (13.0)
Folliculitis	12 (11.3)	0	56 (12.1)
COVID‐19	3 (2.8)	2 (3.8)	47 (10.2)
Pyrexia	0	0	37 (8.0)
Acne	1 (0.9)	1 (1.9)	35 (7.6)
Headache	4 (3.8)	0	30 (6.5)
Eczema	2 (1.9)	0	25 (5.4)
Most common treatment‐related AEs (occurring in ≥2% of patients in the pooled safety population) *n* (%)			
Application site folliculitis	9 (8.5)	0	78 (16.9)
Contact dermatitis	11 (10.4)	0	63 (13.7)
Psoriasis	2 (1.9)	5 (9.6)	37 (8.0)
Folliculitis	7 (6.6)	0	25 (5.4)
Application site pruritus	1 (0.9)	0	14 (3.0)
Acne	0	0	12 (2.6)
Application site irritation	5 (4.7)	1 (1.9)	10 (2.2)
Headache	1 (0.9)	0	9 (2.0)
Most common AEs leading to discontinuation (occurring in ≥1% of patients in the pooled safety population) *n* (%)			
Contact dermatitis	6 (5.7)	0	40 (8.7)
Psoriasis	6 (5.7)	4 (7.7)	19 (4.1)
Dermatitis	1 (0.9)	0	6 (1.3)
Application site pruritus	1 (0.9)	0	5 (1.1)

*Note*: Data are presented as number of patients (%). Summary of adverse event (AEs) in overall trial period of ZBA4‐1 and in ZBA4‐2 is provided in Table S6.

Abbreviations: AEs, adverse events; COVID‐19, coronavirus disease 2019.

^a^ The pooled safety population included the data from ZBA4‐1 and ZBA4‐2 as well as Japanese patients in the tapinarof cream (1%) once daily group of the phase 2 trial (six patients). AEs that occurred in period 1 (vehicle‐treated period) of ZBA4‐1 were excluded from the analyses.

^b^ A serious and severe AE of contact dermatitis in ZBA4‐2 was considered treatment‐related.

In the pooled safety population, AEs were reported in 384 of 461 (83.3%) patients (Table 2). Serious AEs were reported in 2.0% of patients; severe AEs were reported in 1.5% of patients. Of these, a serious and severe AE of contact dermatitis in ZBA4‐2 was considered treatment‐related. The majority of AEs were mild or moderate. The most common AEs included contact dermatitis (18.0%) and application‐site folliculitis (17.6%). Treatment‐related AEs were reported in 51.0% of patients. The most common treatment‐related AEs included application‐site folliculitis (16.9%) and contact dermatitis (13.7%). Trial discontinuations due to AEs occurred in 18.2% of patients. The most common AEs leading to trial discontinuation included contact dermatitis (8.7%) and psoriasis (4.1%). The incidence of AEs did not increase with continued treatment over 52 weeks (Table S7). No clinically significant changes over time were noted in clinical laboratory parameters or vital signs in either trial.

### 3.4 Pharmacokinetics

Across ZBA4‐1 and ZBA4‐2, the plasma concentration of tapinarof was below the LLOQ in approximately 90% of patients at each time point (Table S8). The maximum plasma concentration of tapinarof at each time point ranged from 225 to 1770 pg/mL.

## 4 DISCUSSION

The efficacy results from period 1 of ZBA4‐1 (12‐week double‐blind period) demonstrated that tapinarof cream (1%) was superior to vehicle cream in the treatment of plaque psoriasis. Treatment with tapinarof resulted in improvements in most endpoints by week 12. Across the present trials, improvements in efficacy endpoints continued to increase through week 24 and were maintained through week 52, indicating that tapinarof can be used for long‐term management of plaque psoriasis without loss of efficacy.

Tapinarof was generally safe for up to 52 weeks of treatment. The most common AEs were folliculitis and contact dermatitis, both of which were commonly observed in previous trials of tapinarof in plaque psoriasis.^13^, ^22^, ^23^ In the present trials, all events of folliculitis were mild or moderate; the trial discontinuation rate due to folliculitis was low (<1%). Folliculitis observed with tapinarof can be associated with increased follicular cornification with subsequent plugging, resulting from the upregulation of stratum corneum components, including filaggrin, hornerin, and involucrin.^9^, ^24^ Clinical findings from phase 3 trials of tapinarof in the United States and Canada suggested that folliculitis events were non‐infectious and like keratosis pilaris morphologically.^25^ For contact dermatitis in the present trials, all events were mild or moderate, except for one severe event. Contact dermatitis was the most common AE leading to trial discontinuation in each trial (6.8% to 9.4%). In a dermal safety trial of tapinarof in >200 healthy volunteers, no evidence of contact sensitization was noted under semi‐occlusive conditions.^26^ Reapplication of tapinarof after resolution of tapinarof‐induced contact dermatitis does not uniformly cause recurrence of the dermatitis.^25^ Therefore, Contact dermatitis observed with tapinarof is unlikely to be allergic contact dermatitis. In some patients who developed contact dermatitis, eczematous reactions suggestive of an atopic dermatitis phenotype were seen in the phase 3 trials of tapinarof in the United States and Canada.^25^ These observations may be similar to those reported with biologics for psoriasis, suggesting a transient shift to a T helper 2‐dominated immune response.^27^ In contrast, tapinarof has also been shown to be efficacious in treating atopic dermatitis.^28^, ^29^ The mechanism of tapinarof‐induced contact dermatitis remains to be elucidated.

Limitations of the present two trials include lack of an active comparator; therefore, the superiority of tapinarof over existing therapies for the treatment of psoriasis is uncertain. Further trials are needed to evaluate the efficacy and safety of tapinarof combined with existing therapies. Additionally, given the chronic course of psoriasis, the efficacy and safety of tapinarof remain to be evaluated beyond 52 weeks.

In conclusion, in the 12‐week treatment period, tapinarof was more efficacious than vehicle in Japanese patients with plaque psoriasis. The efficacy response to tapinarof increased with continued treatment and was maintained over 52 weeks. Most AEs were mild or moderate; common AEs included folliculitis and contact dermatitis. Tapinarof was generally safe for up to 52 weeks of treatment. Further investigation is required to provide more information on the positioning of tapinarof in the treatment of plaque psoriasis.

## CONFLICT OF INTEREST STATEMENT

Atsuyuki Igarashi has received advisory board honoraria, consulting fees or speaker honoraria from AbbVie, Eli Lilly Japan, Japan Tobacco, Maruho, Novartis, Sanofi, LEO pharma, and Torii Pharmaceutical; he has also received research grants from AbbVie, Eli Lilly Japan, Japan Tobacco, Novartis, Otsuka Pharmaceutical, Amgen, and Sanofi. Gaku Tsuji has received a research grant and consulting fee from Japan Tobacco. Shuichi Fukasawa, Ryusei Murata, and Satoshi Yamane are employess of Japan Tabacco Inc.

## INFORMED CONSENT STATEMENT

Written informed consent was provided by the patients or the legal guardians of patients aged <20 years.

## CLINICAL TRIAL REGISTRATION NUMBER

The present trials are registered in Japan Registry of Clinical Trials (jRCT), with the registration number of jRCT2031210253 for ZBA4‐1 and jRCT2031210254 for ZBA4‐2.

## Supporting information


Data S1.
