# Supplementary material for: Tapinarof cream for the treatment of plaque psoriasis: Efficacy and safety results from 2 Japanese phase 3 trials
Source: J Dermatol. 2024 Aug 16;51(10):1269–78. doi: 10.1111/1346-8138.17423 (PMC11484133; doi:10.1111/1346-8138.17423)
Supplement: Supplementary file 1 — Data S1. [file JDE-51--s001.pdf]

SUPPORTING INFORMATION

Supplementary Tables

Table S1 Prohibited therapy before baseline (week 0) and during the treatment period.

<table>
<tr><th rowspan="2">Therapy</th><th colspan="2">Washout Period before Week 0</th></tr>
<tr><th>ZBA4-1</th><th>ZBA4-2</th></tr>
<tr><td>IL-12/23p40 inhibitor (ustekinumab),
IL-23p19 inhibitor (e.g., guselkumab)</td><td colspan="2">24 weeks</td></tr>
<tr><td>Other than above, biologic agents that are indicated for psoriasis or that may be effective in psoriasis</td><td colspan="2">12 weeks or 5 half-lives (whichever is longer)</td></tr>
<tr><td>Unapproved drugs in Japan and other investigational products/test drugs, unapproved medical devices in Japan and other investigational devices/test devices</td><td colspan="2">12 weeks or 5 half-lives (whichever is longer)</td></tr>
<tr><td>Phototherapy (can be performed on the hairy scalp)</td><td colspan="2">4 weeks</td></tr>
<tr><td>Systemic treatments that are indicated for psoriasis or that may be effective in psoriasis
Examples: corticosteroids (including inhalant), immunosuppressive agents (e.g., cyclosporin and methotrexate), retinoids, and phosphodiesterase 4 inhibitor (apremilast)
Note: antihistamines and antiallergics (e.g., inhibitors of chemical mediator release) can be used; however, dose and dosing frequency of them should be constant from screening visit.</td><td colspan="2">4 weeks</td></tr>
<tr><td>Live vaccines</td><td colspan="2">4 weeks</td></tr>
<tr><td>Topical corticosteroids classified as strongest or very strong (including combination drugs with vitamin D derivatives) (can be used on the hairy scalp)[a]</td><td colspan="2">2 weeks</td></tr>
<tr><td>Topical treatments that are indicated for psoriasis or that may be effective in psoriasis (can be used on the hairy scalp)
Examples: topical corticosteroids classified as strong or weaker,[a] vitamin D derivatives</td><td>1 week</td><td>from week 0</td></tr>
<tr><td>Moisturizers/protective agents (e.g., heparinoid, urea preparations, petrolatum, and zinc oxide ointment), topical antihistamines, and antiallergics at the application areas of trial treatment
Note: in period 2 of ZBA4-1 and in ZBA4-2, these drugs can be used at the application areas; however, concurrent applications with trial treatment are prohibited.</td><td colspan="2">from week 0</td></tr>
</table>

[a]Topical corticosteroids are classified into 5 ranks (strongest, very strong, strong, medium, and weak) according to the guideline for atopic dermatitis in Japan.[1]

Table S2 Patient demographics and baseline disease characteristics in overall trial period of ZBA4-1 and in the pooled safety population.

	ZBA4-1 Overall trial period[a]		Pooled safety population[c] (n = 461)
	Tapinarof 1% (n = 106)	Vehicle to tapinarof 1%[b] (n = 44)	
Age, mean (SD), years	53.1 (14.0)	60.9 (13.1)	53.8 (13.9)
Male, n (%)	79 (74.5)	40 (90.9)	325 (70.5)
Weight, mean (SD), kg	71.1 (15.5)	70.0 (11.6)	69.8 (14.6)
BMI, mean (SD), kg/m^2	25.4 (4.1)	24.8 (3.3)	25.1 (4.2)
Disease duration, mean (SD), years	13.2 (10.7)	14.5 (9.2)	12.8 (11.3)
PGA score, n (%)			
2: mild	17 (16.0)	7 (15.9)	117 (25.4)
3: moderate	84 (79.2)	35 (79.5)	311 (67.5)
4: severe	5 (4.7)	2 (4.5)	31 (6.7)
PASI score, mean (SD)	10.5 (4.1)	11.0 (4.3)	NC
BSA affected, mean (SD), %	11.7 (5.2)	12.1 (5.4)	12.0 (6.3)
Pruritus NRS score,[d] mean (SD)	3.9 (2.3)	3.5 (2.5)	NC
Skindex-16 total score, mean (SD)	38.6 (20.4)	31.8 (19.3)	NC

Abbreviations: BMI, body mass index; BSA, body surface area; NC, not calculated; NRS, numeric rating scale; PASI, Psoriasis Area and Severity Index; PGA, Physician Global Assessment; SD, standard deviation.

[a]Data based on the efficacy analysis population.

[b]Patients who received vehicle cream in period 1 and received tapinarof cream 1% in period 2.

[c]The pooled safety population included the data from ZBA4-1 and ZBA4-2 as well as Japanese patients in the tapinarof cream 1% once daily group of the phase 2 trial (6 patients).

[d]The baseline value for pruritus NRS score in ZBA4-1 was defined as the mean value of daily scores obtained during 7 days prior to the initiation of trial treatment (Day −7 to Day −1).

Table S3 Summary of rescue therapy.

	ZBA4-1 Overall trial period[a]		ZBA4-2 (n = 304)
	**Tapinarof 1% (n = 106)**	**Vehicle to tapinarof 1%[b] (n = 44)**	
Any rescue medication	5 (4.7)	8 (18.2)	94 (30.9)
Topical corticosteroids[c]	5 (4.7)	6 (13.6)	85 (28.0)
Strongest/very strong[d]	4 (3.8)	2 (4.5)	62 (20.4)
Strong/medium/weak[d]	2 (1.9)	1 (2.3)	25 (8.2)
Topical vitamin D derivatives[e]	1 (0.9)	4 (9.1)	29 (9.5)
Biologic agents	0	0	1 (0.3)
Systemic treatments (except for biologic agents)	1 (0.9)	3 (6.8)	16 (5.3)
Rescue phototherapy	0	0	4 (1.3)

Data are based on the efficacy analysis population for each trial and presented as number of patients (%).

[a]In ZBA4-1, rescue therapy could be used in period 2 only.

[b]Patients who received vehicle cream in period 1 and tapinarof cream 1% in period 2.

[c]Including combination drugs with vitamin D derivatives.

[d]Topical corticosteroids are classified into 5 ranks (strongest, very strong, strong, medium, and weak) according to the guideline for atopic dermatitis in Japan.[1]

[e]Including combination drugs with topical corticosteroids.

Table S4 Summary of efficacy endpoints at week 12, period 1 of ZBA4-1

	Tapinarof 1%	Vehicle	Difference vs. vehicle (95% CI)
**Primary endpoint (MI)[a]**			
PGA treatment success[b]			
n	106	52	
Responder, mean, %	20.06	2.50	18.1 (8.3, 27.9[i])
**Key secondary endpoints (MI)[c]**			
PASI-75[d]			
n	106	52	
Responder, %	37.7	3.8	33.9 (21.3, 44.5[j])
PGA score of 0 or 1			
n	106	52	
Responder, %	30.2	1.9	28.3 (17.1, 38.3[j])
**Other secondary endpoints**			
Change from baseline in PASI score (MI)[e]			
n	106	52	
Mean percent change	−50.56	8.76	−59.32 (−76.72, −41.93)
PASI-50 (MI)[c,d]			
n	106	52	
Responder, %	64.2	13.5	50.7 (35.5, 62.7[j])
PASI-90 (MI)[c,d]			
n	106	52	
Responder, %	14.2	1.9	12.2 (0.7, 20.7[j])
Change from baseline in %BSA affected (MI)[e]			
n	106	52	
Mean change	−2.8	4.3	−7.1 (−9.9, −4.4)
Change from baseline in pruritus NRS score (OC)[f,g]			
n	84	43	
Mean change (SD)	−1.04 (2.50)	0.39 (2.62)	−1.43 (−2.37, −0.49)
≥ 3-point improvement in pruritus NRS score (OC)[f,h]			
n	52	24	
Responder, %	32.7	12.5	20.2 (−2.4, 37.7[j])
≥ 4-point improvement in pruritus NRS score (OC)[f,h]			
n	39	15	
Responder, %	25.6	6.7	19.0 (−9.0, 37.5[j])

	Tapinarof 1%	Vehicle	Difference vs. vehicle (95% CI)
Change from baseline in Skindex-16 score (OC)[f]			
n	84	43	
Total score, mean change (SD)	−14.14 (23.58)	0.36 (22.47)	−14.50 (−23.11, −5.89)
Symptom subscale score, mean change (SD)	−8.78 (30.68)	4.94 (28.50)	−13.72 (−24.84, −2.60)
Emotional subscale score, mean change (SD)	−22.93 (30.56)	−3.60 (25.36)	−19.33 (−30.06, −8.60)
Functional subscale score, mean change (SD)	−6.11 (20.49)	2.25 (20.38)	−8.36 (−15.95, −0.77)

Abbreviations: BSA, body surface area; CI, confidence interval; MI, multiple imputation; NRS, numeric rating scale; OC, observed cases; PASI, Psoriasis Area and Severity Index; PGA, Physician Global Assessment; SD, standard deviation.

[a]The primary endpoint was analyzed on the basis of 100 datasets where missing data were imputed by the MI.

[b]PGA treatment success was defined as a PGA score of 0 or 1 with ≥ 2-grade improvement from baseline.

[c]These endpoints were analyzed on the basis of the first dataset out of 100 datasets where missing data were imputed by the MI.

[d]PASI-50, -75, and -90 were defined as ≥ 50%, ≥ 75%, and ≥ 90% improvement from baseline in PASI score, respectively.

[e]These endpoints were analyzed on the basis of the data imputed by the MI.

[f]These endpoints were analyzed on the basis of OC where missing data were not imputed.

[g]The baseline value for pruritus NRS score was defined as the mean value of daily scores obtained during 7 days prior to the initiation of trial treatment (Day −7 to Day −1). The pruritus NRS score at week 12 was defined as the mean value of daily scores obtained during 7 days prior to the visit.

[h]The analyses of ≥ 3-point and ≥ 4-point improvement in pruritus NRS score were performed in patients who had a baseline pruritus NRS score of ≥ 3 and ≥ 4, respectively.

[i]Sato method.[2]

[j]Exact 95% CI.

Table S5 Summary of efficacy endpoints by trial visit

	ZBA4-1 (overall trial period)				ZBA4-2	
	n	Tapinarof 1%	n	Vehicle to Tapinarof 1%[a]	n	Tapinarof 1%
PGA treatment success[b] rate, % (exact 95% CI)						
Week 4	92	4.3 (1.2, 10.8)	41	0 (0.0, 8.6)	282	5.0 (2.7, 8.2)
Week 12	84	19.0 (11.3, 29.1)	43	2.3 (0.1, 12.3)	260	30.0 (24.5, 36.0)
Week 24	77	58.4 (46.6, 69.6)	37	45.9 (29.5, 63.1)	236	51.3 (44.7, 57.8)
Week 52		NA		NA	224	56.3 (49.5, 62.8)
Patients with a PGA score of 0 or 1, % (exact 95% CI)						
Week 4	92	7.6 (3.1, 15.1)	41	0 (0.0, 8.6)	282	7.4 (4.7, 11.2)
Week 12	84	28.6 (19.2, 39.5)	43	2.3 (0.1, 12.3)	260	38.1 (32.1, 44.3)
Week 24	77	64.9 (53.2, 75.5)	37	48.6 (31.9, 65.6)	236	63.6 (57.1, 69.7)
Week 52		NA		NA	224	69.6 (63.2, 75.6)
Mean percent change from baseline in PASI score, % (SD)						
Week 4	92	−40.30 (33.89)	41	6.10 (47.58)	282	−40.56 (33.81)
Week 12	83	−56.19 (41.23)	43	3.37 (54.02)	260	−67.21 (32.72)
Week 24	77	−78.49 (56.49)	37	−67.89 (48.00)	236	−80.55 (29.27)
Week 52		NA		NA	224	−85.03 (20.66)
PASI-50[c] response rate, % (exact 95% CI)						
Week 4	92	47.8 (37.3, 58.5)	41	4.9 (0.6, 16.5)	282	46.8 (40.9, 52.8)
Week 12	83	71.1 (60.1, 80.5)	43	16.3 (6.8, 30.7)	260	77.7 (72.1, 82.6)
Week 24	77	92.2 (83.8, 97.1)	37	81.1 (64.8, 92.0)	236	90.7 (86.2, 94.1)
Week 52		NA		NA	224	95.5 (91.9, 97.8)
PASI-75[c] response rate, % (exact 95% CI)						
Week 4	92	18.5 (11.1, 27.9)	41	0 (0.0, 8.6)	282	16.3 (12.2, 21.2)
Week 12	83	42.2 (31.4, 53.5)	43	4.7 (0.6, 15.8)	260	50.4 (44.1, 56.6)
Week 24	77	80.5 (69.9, 88.7)	37	64.9 (47.5, 79.8)	236	77.5 (71.7, 82.7)
Week 52		NA		NA	224	79.9 (74.1, 85.0)
PASI-90[c,d] response rate, % (exact 95% CI)						
Week 4	92	1.1 (0.0, 5.9)	41	0 (0.0, 8.6)	282	2.8 (1.2, 5.5)
Week 12	83	15.7 (8.6, 25.3)	43	2.3 (0.1, 12.3)	260	23.5 (18.4, 29.1)
Week 24	77	54.5 (42.8, 65.9)	37	32.4 (18.0, 49.8)	236	47.5 (40.9, 54.0)
Week 52		NA		NA	224	55.4 (48.6, 62.0)
Mean change from baseline in %BSA affected (SD)						
Week 4	92	−1.6 (3.7)	41	2.3 (7.1)	282	−1.4 (4.6)
Week 12	83	−3.6 (6.7)	43	3.5 (9.1)	260	−5.0 (6.4)
Week 24	77	−8.4 (7.8)	37	−4.9 (8.2)	236	−7.9 (6.8)
Week 52		NA		NA	224	−9.2 (5.9)

	ZBA4-1 (overall trial period)				ZBA4-2	
	n	Tapinarof 1%	n	Vehicle to Tapinarof 1%[a]	n	Tapinarof 1%
Mean change from baseline in pruritus NRS score[e] (SD)						
Week 4	92	−0.88 (2.31)	41	0.50 (2.40)	282	−0.5 (2.9)
Week 12	84	−1.04 (2.50)	43	0.39 (2.62)	260	−1.8 (3.1)
Week 24	77	−2.48 (2.72)	37	−1.33 (2.98)	236	−2.7 (2.9)
Week 52		NA		NA	224	−3.1 (2.7)
Mean change from baseline in Skindex-16 total score (SD)						
Week 4	92	−11.90 (18.64)	41	−0.33 (18.12)	282	−10.13 (18.24)
Week 12	84	−14.14 (23.58)	43	0.36 (22.47)	260	−17.50 (21.64)
Week 24	77	−24.78 (22.15)	37	−14.10 (18.98)	236	−24.92 (20.50)
Week 52		NA		NA	224	−27.22 (19.11)

Abbreviations: BSA, body surface area; CI, confidence interval; NA, not applicable; NRS, numeric rating scale; PASI, Psoriasis Area and Severity Index; PGA, Physician Global Assessment; SD, standard deviation.

All efficacy endpoints were analyzed on the basis of observed cases (OC) where missing data were not imputed.

[a]In the vehicle to tapinarof group of ZBA4-1, patients switched from vehicle cream to tapinarof cream 1% at week 12.

[b]PGA treatment success was defined as a PGA score of 0 or 1 with ≥ 2-grade improvement from baseline.

[c]PASI-50, -75, and -90 were defined as ≥ 50%, ≥ 75%, and ≥ 90% improvement from baseline in PASI score, respectively.

[d]PASI-90 in overall trial period of ZBA4-1 was analyzed post hoc.

[e]In ZBA4-1, the baseline value for pruritus NRS score was defined as the mean value of daily scores obtained during 7 days prior to the initiation of trial treatment (Day −7 to Day −1). The pruritus NRS score at each visit from week 2 to week 12 (period 1) was defined as the mean value of daily scores obtained during 7 days prior to the relevant visit, and the scores from week 16 to week 24 (period 2) were assessed at each visit. In ZBA4-2, the pruritus NRS scores were assessed at each visit throughout the trial.

Table S6 Summary of adverse events in overall trial period of ZBA4-1 and in ZBA4-2.

	**ZBA4-1 Overall trial period**		**ZBA4-2 (n = 304)**
	**Tapinarof 1% (n = 106)**	**Vehicle to tapinarof[a] (n = 44)**	
Any AEs	82 (77.4)	22 (50.0)	277 (90.8)
Serious AEs[b]	0	0	9 (3.0)
Severe AEs[b]	1 (0.9)	0	6 (2.0)
Treatment-related AEs	54 (50.9)	12 (27.3)	169 (55.4)
AEs leading to discontinuation	22 (20.8)	5 (11.4)	57 (18.7)
Most common AEs (occurring in ≥ 5% of patients in the pooled safety population)			
Contact dermatitis	18 (17.0)	8 (18.2)	57 (18.7)
Application site folliculitis	17 (16.0)	5 (11.4)	59 (19.3)
Psoriasis	11 (10.4)	3 (6.8)	46 (15.1)
Folliculitis	16 (15.1)	0	40 (13.1)
COVID-19	4 (3.8)	0	43 (14.1)
Pyrexia	1 (0.9)	1 (2.3)	35 (11.5)
Acne	3 (2.8)	1 (2.3)	31 (10.2)
Headache	4 (3.8)	0	26 (8.5)
Eczema	3 (2.8)	0	22 (7.2)
Most common treatment-related AEs (occurring in ≥ 2% of patients in the pooled safety population)			
Application site folliculitis	15 (14.2)	5 (11.4)	58 (19.0)
Contact dermatitis	14 (13.2)	5 (11.4)	44 (14.4)
Psoriasis	5 (4.7)	2 (4.5)	30 (9.8)
Folliculitis	8 (7.5)	0	17 (5.6)
Application site pruritus	2 (1.9)	0	12 (3.9)
Acne	0	0	12 (3.9)
Application site irritation	6 (5.7)	0	4 (1.3)
Headache	1 (0.9)	0	8 (2.6)
Most common AEs leading to discontinuation (occurring in ≥ 1% of patients in the pooled safety population)			
Contact dermatitis	10 (9.4)	3 (6.8)	27 (8.9)
Psoriasis	6 (5.7)	1 (2.3)	12 (3.9)
Dermatitis	3 (2.8)	0	3 (1.0)
Application site pruritus	2 (1.9)	0	3 (1.0)

Abbreviations: AE, adverse event; COVID-19, coronavirus disease 2019.

Data are presented as number of patients (%).

[a]Patients who received vehicle cream in period 1 and received tapinarof cream 1% in period 2. Adverse events that occurred in period 1 were excluded from the analyses.

[b]A serious and severe AE of contact dermatitis in ZBA4-2 was considered treatment-related.

Table S7 Adverse events by time of onset occurring in ≥ 5% of patients in the pooled safety population.

	Time of onset				
	**Week 0 to Week 12[a] (n = 461)**	**Week 12 to Week 24[b] (n = 401)**	**Week 24 to Week 36[c] (n = 322)**	**Week 36 to Week 48[d] (n = 235)**	**Week 48 to Week 52[e] (n = 231)**
Any AEs	272 (59.0)	206 (51.4)	120 (37.3)	107 (45.5)	35 (15.2)
Contact dermatitis	53 (11.5)	29 (7.2)	6 (1.9)	6 (2.6)	3 (1.3)
Application site folliculitis	52 (11.3)	24 (6.0)	9 (2.8)	8 (3.4)	1 (0.4)
Psoriasis	40 (8.7)	14 (3.5)	5 (1.6)	5 (2.1)	2 (0.9)
Folliculitis	26 (5.6)	18 (4.5)	13 (4.0)	5 (2.1)	1 (0.4)
COVID-19	7 (1.5)	8 (2.0)	10 (3.1)	18 (7.7)	5 (2.2)
Pyrexia	11 (2.4)	18 (4.5)	5 (1.6)	4 (1.7)	1 (0.4)
Acne	5 (1.1)	13 (3.2)	12 (3.7)	6 (2.6)	3 (1.3)
Headache	23 (5.0)	6 (1.5)	4 (1.2)	1 (0.4)	0
Eczema	13 (2.8)	6 (1.5)	3 (0.9)	3 (1.3)	1 (0.4)

Abbreviations: AE, adverse event; COVID-19, coronavirus disease 2019.

Data are presented as number of patients (%).

The pooled safety population included the data from ZBA4-1 and ZBA4-2 as well as Japanese patients in the tapinarof cream 1% once daily group of the phase 2 trial (6 patients). Adverse events that occurred in period 1 (vehicle-treated period) of ZBA4-1 were excluded from the analyses.

The denominator used for calculation of percentage was the number of patients in each period who remained in the trials.

When a patient had ≥ 2 events of the same preferred term in different periods, the patient was counted in each period.

[a]Day 1 to Day 77, [b]Day 78 to Day 161, [c]Day 162 to Day 245, [d]Day 246 to Day 329, [e]Day 330 and later

Table S8 Summary of plasma concentrations of tapinarof.

	Week 4	Week 12	Week 24	Week 52
**ZBA4-1 (overall trial period)**				
Tapinarof 1%				
Number of patients	92	87	77	--
Patients with a ≥ LLOQ value, n (%)	11 (12.0)	14 (16.1)	11 (14.3)	--
Mean plasma concentration (SD), pg/mL	19.90 (70.37)	33.93 (122.2)	37.50 (206.4)	--
Maximum plasma concentration, pg/mL	508	776	1770	--
Vehicle to Tapinarof 1%				
Number of patients	--	--	37	--
Patients with a ≥ LLOQ value, n (%)	--	--	3 (8.1)	--
Mean plasma concentration (SD), pg/mL	--	--	11.19 (42.58)	--
Maximum plasma concentration, pg/mL	--	--	225	--
**ZBA4-2**				
Number of patients	288	259	240	224
Patients with a ≥ LLOQ value, n (%)	36 (12.5)	26 (10.0)	10 (4.2)	4 (1.8)
Mean plasma concentration (SD), pg/mL	18.04 (72.22)	18.52 (90.56)	5.922 (35.52)	2.718 (23.18)
Maximum plasma concentration, pg/mL	743	1070	368	287

Abbreviations: LLOQ, lower limit of quantification; SD, standard deviation.

For the analysis of plasma concentration, values < LLOQ (50 pg/mL) were treated as 0.

Supplementary Figures

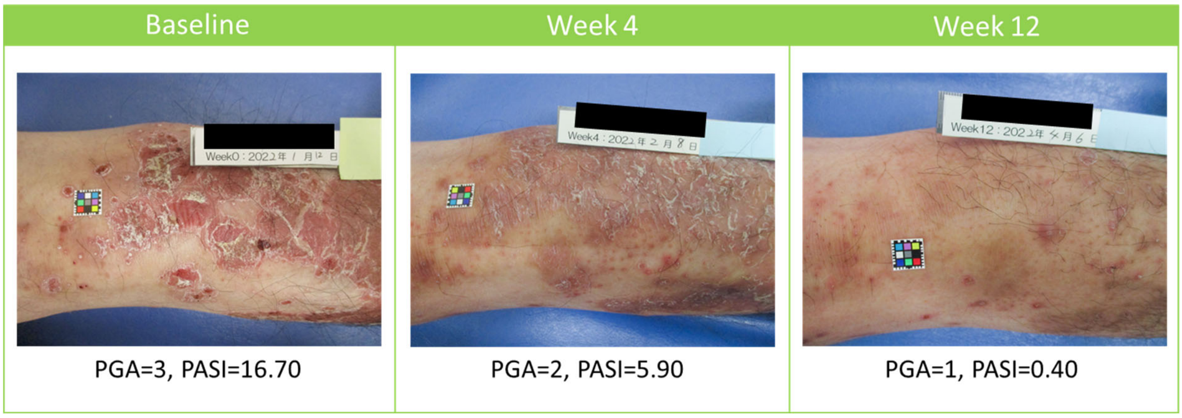

Patient 1, knee

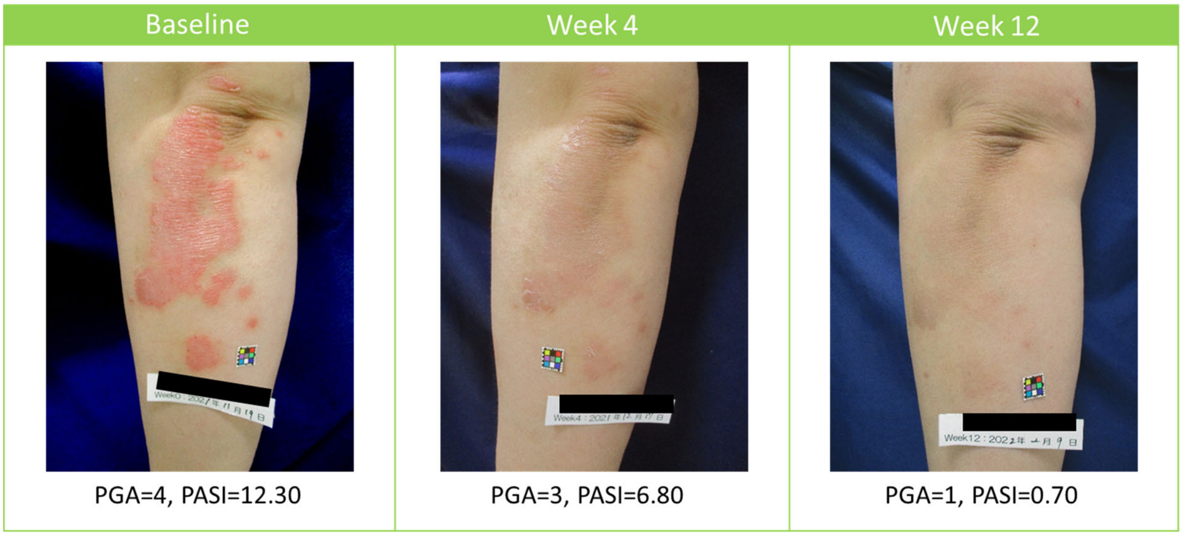

Patient 2, forearm

Figure S1 Representative clinical images of patients receiving tapinarof who achieved PGA treatment success in period 1 of ZBA4-1. The PGA and PASI scores below the images are reflective of not the presented lesions but the entire body. PASI, Psoriasis Area and Severity Index; PGA, Physician Global Assessment. PGA treatment success was defined as a PGA score of 0 or 1 with ≥ 2-grade improvement from baseline.

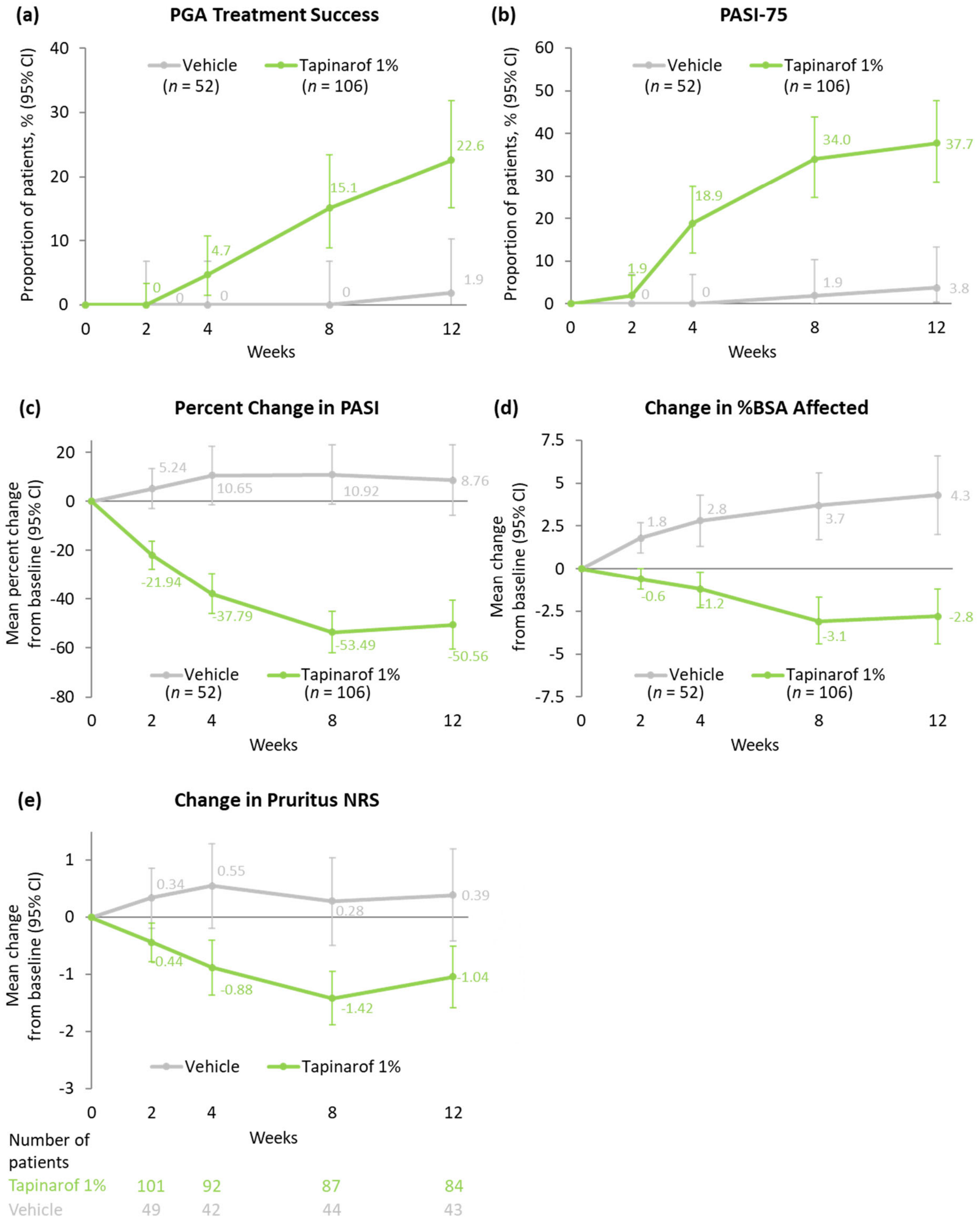

Figure S2 Efficacy endpoints by trial visit in period 1 of ZBA4-1. (a) PGA treatment success rate, (b) PASI-75 response rate, (c) percent change from baseline in PASI score, (d) change from baseline in %BSA affected, and (e) change from baseline in pruritus NRS score. BSA, body surface area; CI, confidence interval; NRS, numeric rating scale; PASI, Psoriasis Area and Severity Index; PGA, Physician Global Assessment. PGA treatment success was defined

as a PGA score of 0 or 1 with ≥ 2-grade improvement from baseline. PASI-75 was defined as ≥ 75% improvement from baseline in PASI score. The pruritus NRS score at each visit was defined as the mean value of daily scores obtained during 7 days prior to the relevant visit. (a) and (b) were based on the first dataset out of 100 datasets where missing data were imputed by the multiple imputation (MI) are presented with exact 95% CIs. (c) and (d) were based on the data imputed by the MI. (e) was based on observed cases (OC) where missing data were not imputed.

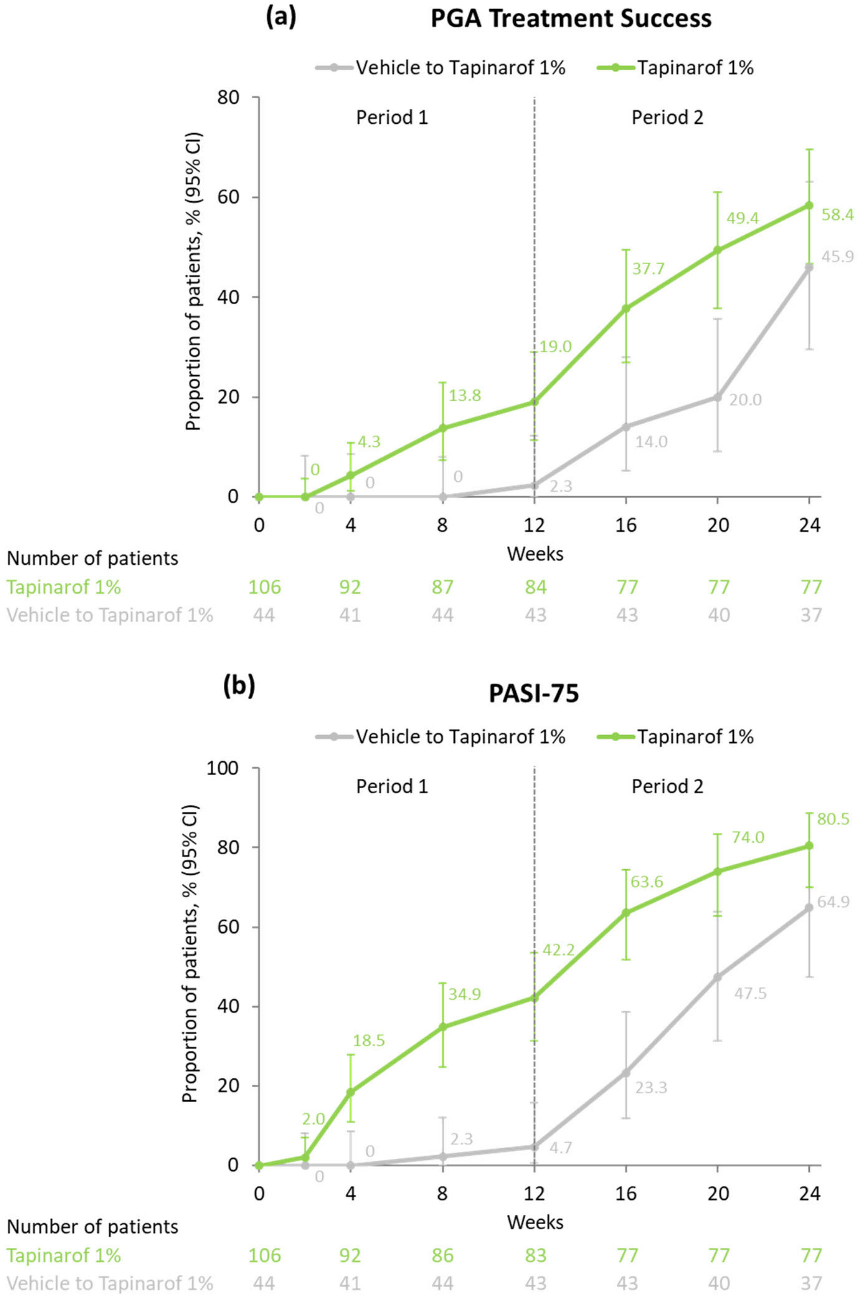

Figure S3 Proportion of patients with (a) PGA treatment success and (b) PASI-75 in overall trial period of ZBA4-1. CI, confidence interval; PASI, Psoriasis Area and Severity Index; PGA, Physician Global Assessment. PGA treatment success was defined as a PGA score of 0 or 1 with ≥ 2-grade improvement from baseline. PASI-75 was defined as ≥ 75% improvement from baseline in PASI score. In the vehicle to tapinarof group, patients switched from vehicle cream to tapinarof cream 1% at week 12. Vehicle-treated patients who were discontinued from the trial in period 1 (by week 12) were excluded from the analyses. Data were analyzed on the basis of observed cases (OC) where missing data were not imputed and are presented with exact 95% CIs.

References for Supporting Information

1. Saeki H, Ohya Y, Furuta J, Arakawa H, Ichiyama S, Katsunuma T, et al. English Version of Clinical Practice Guidelines for the Management of Atopic Dermatitis 2021. J Dermatol. 2022 Oct;49(10):e315-e375. doi: 10.1111/1346-8138.16527.

2. Sato T, Greenland S, Robins JM. On the Variance Estimator for the Mantel-Haenszel Risk Difference. Biometrics. 1989 45(4):1323-1324. doi: 10.2307/2531784
